# Maximizing oil recovery: Innovative chemical EOR solutions for residual oil mobilization in Kazakhstan's waterflooded sandstone oilfield

**DOI:** 10.1016/j.heliyon.2024.e28915

**Published:** 2024-03-28

**Authors:** Mariam Shakeel, Dilyara Sagandykova, Aibek Mukhtarov, Amina Dauyltayeva, Laila Maratbekkyzy, Peyman Pourafshary, Darya Musharova

**Affiliations:** a School of Mining and Geosciences, Nazarbayev University, Astana, 010000, Kazakhstan; b KazMunayGas Engineering, Astana, 010017, Kazakhstan

## Abstract

The results of an experimental study to design a chemical flood scheme for a massive Kazakhstani oilfield with high water cut are presented in this paper. A meticulously formulated chemical flooding procedure entails injecting a blend comprising interfacial tension (IFT) reducing agents, alkaline/nanoparticles to control chemical adsorption, and polymer to facilitate mobility control. Overall, this well-conceived approach leads to a significant enhancement in the mobilization and production of residual oil.

Experiments were conducted in Kazakhstan's Field A, one of the country's oldest oilfields with over 90% water cut and substantial remaining oil, to assess the efficiency of various hydrolyzed polyacrylamide (HPAM) derived polymers and surfactant solutions. Additionally, the effectiveness of alkaline and nanoparticles in minimizing chemical adsorption for the screened surfactant and polymer was investigated. These assessments were conducted under reservoir conditions, with a temperature of 63 °C, and using 13,000 ppm Caspian seawater as makeup brine.

The performance assessment of the selected chemicals was carried out through a set of oil displacement tests on reservoir cores. Critical parameters, including chemical adsorption, interfacial tension, resistance factor, and oil recovery factor, were compared to determine the most effective chemical flooding approach for Field A. Both the surfactant-polymer (SP) and alkali-surfactant-polymer (ASP) approaches were more successful in recovering residual oil by efficiently generating and delivering microemulsion, producing more than 90% of the remaining oil after waterflooding. Due to the low increase in recovery compared to SP and the complexity of applying ASP at the field scale, SP was recommended for the pilot test studies.

This investigation underscores that the choice of chemicals is contingent upon the interplay between the specific characteristics of the oil, the geological formation, the injection water, and the reservoir rock. Consequently, assessing all potential configurations on reservoir cores is imperative to identify the most optimal chemical combination. The practical challenges at the field scale should also be considered for the final decision. The results of this study contribute to the successful design and implementation of tailored chemical flooding to challenging oilfields with excessive water cut and high residual oil.

## 1 Introduction

Oil extraction involves three main stages: primary, secondary, and tertiary. Primary production utilizes natural reservoir energy to extract around 10–25% of the original oil in place (OOIP). Secondary methods, like water or gas flooding, introduce fluids to maintain pressure and displace oil, leaving a significant amount unrecovered. The low recovery coefficient is primarily caused by the unfavorable mobility ratio between the injected fluid and the existing crude oil. Moreover, hydrocarbons are either bypassed by the injected water or are still trapped in small pores, resulting in the widespread challenge of high water cut and lower recovery factor [1,2]. The oil and gas industry relies on enhanced oil recovery (EOR) methods to boost oil production and access untapped reserves. Among EOR methods, chemical methods stand out for their ability to modify reservoir conditions and fluid properties. Alkaline, surfactant, polymer, and nanoparticle flooding are key chemical EOR techniques. They improve the oil recovery by enhancing fluid-rock interactions, reducing interfacial tension, increasing fluid viscosity, altering wettability, and improving sweep efficiency. When applied effectively, they contribute to the successful recovery of unrecovered oil reserves and maximize hydrocarbon production from reservoirs.

By injecting polymer solutions into reservoirs, polymer flooding significantly raises the viscosity of the injected fluid [3]. A viscous displacement front is formed by highly viscous polymer solutions, which causes fluid flow to be diverted from high-permeability paths and into previously unswept or low-permeability areas. This method lowers the mobility ratio between injected fluid and oil by mobilizing any leftover oil after primary and secondary extraction [4]. Higher polymer concentrations are maintained in the liquid phase due to changes in wettability and decreased polymer adsorption caused by contact with reservoir rock. This method has been successfully implemented in numerous oil fields worldwide, including sandstone reservoirs with high water cut and viscous/heavy oil [1,5,6].

Flooding with surfactants improves oil recovery by reducing interfacial tension and emulsification. Surfactants reduce the interfacial tension between oil and water and increase the ability of water or other displacing fluids to contact and recover oil [7]. Moreover, surfactants can form emulsions by dispersing oil in water or vice versa. Emulsification involves the formation of small oil droplets within the water phase, with droplet sizes typically equal to or smaller than the pore sizes [8]. Due to the efficient movement of tiny droplets through the reservoir, these emulsions increase oil mobility. Larger droplets, on the other hand, may cause pore plugging and entrapment, which will drive the surfactant solution to unswept sections of the porous media and boost macroscopic sweep efficiency [4]. These mechanisms contribute to the enhanced recovery of oil and the improved displacement of oil from the reservoir.

The combination of polymer flooding and surfactant flooding forms one of the most popular hybrid chemical EOR methods that has gained more attention in recent years. SP flooding combines the benefits of each technique: polymers are utilized to raise the viscosity of the injected water, creating a more favorable mobility ratio, while surfactants are employed to reduce the interfacial tension between oil and water [9,10]. To minimize surfactant adsorption, the primary approach involves injecting polymers as sacrificial agents prior to introducing surfactants. On the other hand, if the primary goal is to adjust the mobility ratio, polymers are injected following surfactant slugs [4].

Surfactant flooding frequently includes the addition of alkalis to increase effectiveness. These alkaline agents serve as sacrificial components, inhibiting the adsorption of surfactants on rock surfaces, and interact with the acidic groups in crude oil to produce in-situ soaps that result in the same effect as flooding with surfactants [11,12]. Polymer flooding can be used with alkali flooding as well. While alkaline flooding has shown potential for enhancing oil recovery, field applications have revealed instances where the incremental oil recovery was not significantly greater than conventional waterflooding. This may be because they lack mobility control [4]. To overcome this limitation, one option is to incorporate polymers as mobility control agents, thereby improving the efficiency of alkaline treatment. Table 1 presents a comparison of mechanisms involved in different chemical EOR techniques.

**Table 1:** Comparison of mechanisms involved in different chemical EOR techniques.

Type	Mechanism(s)
Polymer flooding [13]	• Mobility control
Surfactant-polymer flooding [14]	• IFT reduction • Mobility control
Alkaline-surfactant-polymer flooding [15]	• Surfactant and polymer adsorption reduction • In situ surfactant generation • Ultra-low IFT • Mobility control
Nano-surfactant-polymer flooding [16,17]	• Surfactant and polymer adsorption reduction • IFT reduction • Polymer viscosity enhancement • Mobility control • Wettability alteration

Alkali-surfactant-polymer (ASP) flooding, recognized as the most promising hybrid chemical EOR method, provides a greater increase in oil recovery compared to chemical flooding involving only one or two components [2,4]. By injecting three chemical agents in the proper sequence, the integrated synergy of these components is fully harnessed. Polymers enhance sweep efficiency, surfactants reduce interfacial tension (IFT), and alkalis generate in-situ surfactants. Interfacial tension is a measure of the force per unit length acting at the interface between two immiscible phases, such as oil-water interface. IFT plays a decisive part in numerous phenomena, particularly the stability of emulsions. Moreover, ASP flooding helps overcome individual component drawbacks such as poor mobility control, high alkali consumption, and excessive surfactant adsorption [18,19].

When added to these solutions, nanoparticles (NPs) effectively improve surfactant or polymer flooding, resulting in significantly more oil recovery [20]. NPs are being investigated as a potential solution to prevent polymer damage during polymer flooding. A significant barrier to efficiency is overcome when nanoparticles and surfactants are mixed because this reduces surfactant adsorption on reservoir rocks [21]. This treatment offers several benefits, such as improving rheological properties, thermal stability, and salinity and pressure tolerance of polymers, reduced IFT by synergy of NPs and surfactants, and altered wettability by NPs [22]. NPs have been employed in several applications to minimize polymer and surfactant adsorption. Steric hindrance is a key process in which NPs physically block polymer chains or surfactant molecules from reaching surfaces or interfaces [[23], [24], [25]]. NPs may also produce electrostatic repulsion, especially when their surface charges interact with charged polymer or surfactant molecules. This interaction results in repulsion, leading to a decrease in adsorption. NPs can also limit adsorption by forming surface barriers that prevent molecules from interacting or reduce their surface attraction. Hydrophobic NPs may disrupt surfactant adsorption by interacting with hydrophobic regions, resulting in aggregates [26]. NPs' small size and unique properties may affect their interactions with polymers and surfactants. The selection of NPs and surface modifications must be tailored to each application since the success of these procedures is linked to brine composition and type of rock [27,28].

As per recent research findings, NPs have demonstrated a notable capacity to reduce the adsorption of surfactants on porous materials [[29], [30], [31], [32], [33], [34], [35]]. Notably, Wu et al. observed a significant drop in the adsorption density of anionic surfactants on the surfaces of quartz sand. This drop was substantial, declining from 2.84 mg/g to 1.61 mg/g after introducing 0.5 wt% SiO_2_ NPs to the surfactant solution. The research indicated that surfactant molecules preferred attaching to the NPs rather than binding to the sand, with the NPs essentially serving as a protective barrier on the sand surface. Furthermore, during surfactant/nanofluid flooding, the vigorous interactions and friction between NPs and the sand surface dislodged the surfactant molecules that were previously adsorbed, resulting in the reduction of surfactant adsorption [31]. Similarly, Zhong et al. found a 20.1% and 40.9% reduction in non-ionic surfactant adsorption on Bakken and Berea samples upon incorporating SiO_2_ NPs. The favorable impact of NPs is contingent on the characteristics of the adsorbent. The researchers proposed that this variation might be linked to the relatively higher presence of clay and carbonate in the Bakken samples, which trap SiO_2_ NPs, ultimately leading to increased adsorption density [30].

In another study, separate static adsorption experiments indicated that NPs could decrease polymer adsorption on rock. Because NPs have unique surface characteristics, such as electrostatic properties, adsorption may be reduced. When 1 wt% alkali and 0.08 wt% polymer were used, the adsorption decreased to 58.45, 53.12, and 47.23 mg/g at 0, 0.05, and 0.1 wt% silica NPs, respectively. This reduction can be attributed to the nanoparticle-polymer interaction and the repulsion between the charged surfaces, resulting in decreased polymer adsorption on the rock [36].

Field A in Kazakhstan is a potential candidate to evaluate and propose using the above-mentioned methods to increase oil recovery.

The reported chemical enhanced oil recovery techniques heavily depend on capillary desaturation, a crucial component of reservoir dynamics. Capillary forces control the flow and distribution of fluids in porous medium in reservoirs. Capillary desaturation happens as primary and secondary extraction techniques are used, leaving trapped oil in tiny pores because of capillary pressure that opposes fluid flow [37]. By modifying the capillary forces, chemical EOR approaches like polymer flooding, surfactant flooding, and their mixtures like ASP flooding strategically target these trapped oil spots. Thus, the capillary desaturation curve (CDC), also known as the connection between residual oil saturation and capillary number, is the theoretical foundation for these chemical EOR applications. The primary finding of various CDCs is that various chemicals should be used to achieve the lowest possible residual oil saturation and the highest possible displacement efficiency [38,39].

In EOR, the link between capillary desaturation and interfacial tension reduction holds significant importance, particularly in surfactant-polymer (SP) flooding. Surfactants are critical in reducing the interfacial tension between oil and water, facilitating oil separation from rock surfaces [40,41]. With decreased interfacial tension, capillary desaturation becomes increasingly influential, enhancing oil displacement and improved recovery. This interplay between capillary desaturation and the reduction of interfacial tension underscores the necessity of understanding these factors to optimize strategies for SP flooding, aiming at efficient and sustainable oil recovery [42,43].

Field A in Kazakhstan is a sandstone oilfield containing waxy oil, with permeability and porosity of 100–500 md and 24–27%, respectively. The crude oil in Field A has a density of approximately 0.80 g/cc and a viscosity of 4.5 cp at a reservoir temperature of 63 °C. It also contains 13–15 wt% asphaltenes and 20–28 wt% paraffin content. Additionally, the formation water in Field A has a high salinity of 120,000 ppm, with a significant concentration of monovalent and divalent ions.

Early oil extraction began in 1967 when Field A was flooded by cold Caspian seawater (SW), which created a problem of asphaltene precipitation [44]. Firstly, untreated cold seawater solidified paraffin, blocked oil channels, and clogged injectors. Plugged areas led to increased water production. Due to seawater salts' interaction with formation oil, secondary EOR produced corrosive deposits that contaminated surfaces and subterranean structures [44,45]. After that, a hot water injection project led to the development of a more successful strategy by lowering the oil viscosity and reducing the risk of asphaltene and wax deposition. Hot water injection at 85–90 °C produced controlled water production and an oil recovery of 34–38% [46,47]. In 1975, the oil industry reached its peak production level of 16.249 million tons. However, there was a huge increase in water cut from 24% to 53.4% and a significant reduction in oil production of 35.5% between 1976 and 1983. From 1991 to 1999, the field's development became unstable, leading to a noticeable increase in injected water and the most significant increase in water cut during its development [46]. The most important fact is that currently, Field A is encountering a significant challenge of high water cut, which has reached 90% across the entire field. The field still has a high residual oil saturation of more than 50%. The purpose of this article is to determine the best chemical EOR method that has the potential to improve oil production in Field A by effectively managing the water cut and mobilizing the residual oil.

In particular, the most common and widely applied solution for high water cuts is polymer flooding [48,49]. The examples of several oil fields where polymer flooding was successful are shown in Table 2.

**Table 2:** Summary of oil fields subject to polymer flooding.

Field	Properties	Result
Daqing Field, China [50]	Lithology – sandstone; Density – from 33 to 39°API; Depth – from 900 to 1200 m; Permeability – from 500 to 1000 mD; Porosity – from 25 to 30%; High wax content.	The total incremental oil production by polymer flooding alone represented 6.03 million tons.
Bhagyam Field, Rajasthan [51,52]	Lithology – sandstone; Viscosity – from 15 to 20 cP; Permeability – from 1000 to 10000 mD; Porosity – from 25 to 30%.	Polymer injection led to a 1–35% water cut reduction in all producers and raised the oil production rate from 10500 to 15500 BPD.
Al Khalata reservoir, Sultanate of Oman field [53]	Lithology – sandstone; Density – 22°API; Viscosity – 90 cP; Permeability – from 100 to 2000 mD; Porosity – from 25 to 30%.	The water cut drop is between 2 and 30% and the increase in oil production is about 25%.

However, adsorption of polymer molecules onto rock surface is a constraint of polymer flooding that reduces the injection's effectiveness by lowering the polymer concentration in-situ. To gain the maximum possible incremental oil recovery, polymer flooding can be combined with other techniques in light of the above-mentioned synergies of surfactant, alkali, and NPs.

A systematic oil displacement study was required to select a suitable EOR design for Field A. The goal of this study was to ascertain whether compounds that have been selected using various static methods and screening criteria increase oil output. Various EOR techniques—such as polymer, surfactant, alkaline, and nanoparticle flooding, and their possible combinations—were assessed based on their capacity to displace oil and increase recovery factor in porous medium. By focusing on oil displacement results, the primary objective of this research was to determine the most effective and novel EOR method for Field A that would benefit from the synergy among different chemicals.

The novelty of this design study on chemical EOR for Field A is evident in its customized approach to tackle the distinct geological and reservoir conditions specific to the region. The study's innovation lies in its customization, which involves integrating field reservoir samples and conducting thorough analyses at various scales. Through the integration of field reservoir samples, including crude oil, formation water, and seawater, and the implementation of thorough bulk-scale, micro-scale, and interfacial analyses, the study sought to create a custom-made chemical EOR solution tailored for the intricate subsurface conditions characterized by high temperature, elevated salinity, bacterial presence, and waxy oil with a substantial paraffin content in the target oil field. Furthermore, the study embraced inventive formulations of chemicals, encompassing carefully designed blends of surfactants, polymers, alkaline agents, and NPs, to augment oil mobility and improve reservoir sweep efficiency. The recognition of the field's specific challenges, such as high salinity (∼60,000 ppm) and temperature (63 °C), distinguishes this design study, highlighting a commitment to surmounting site-specific obstacles and optimizing the effectiveness of the chemical EOR method. This distinctive and pioneering approach establishes the study as a trailblazing initiative in advancing sustainable and efficient oil recovery practices within the Kazakhstan oil industry.

## 2 Methodology

**Fig. 1:**
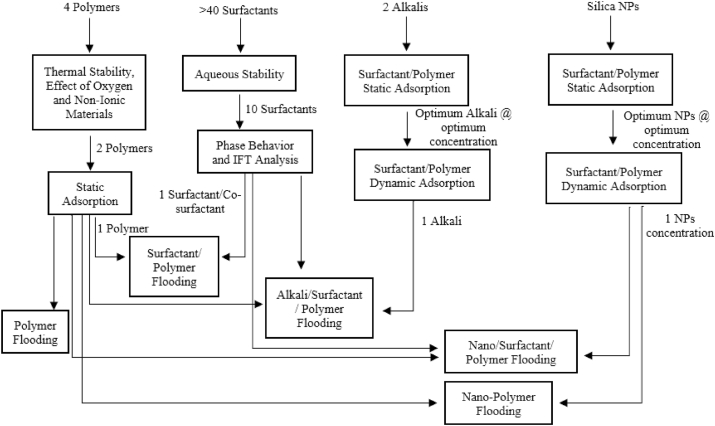
Flow chart showing the experiments and their sequence followed during the study.

The methodology adopted in this study comprised several preliminary and intermediate tests to screen the best chemicals from the available options. To start with, 4 polymers, more than 40 surfactant mixtures, 2 alkalis, and silica NPs were passed through a series of screening tests. A step-wise systematic flow of experiments carried out during the study is given in Fig. 1. In the first stage, polymer screening was carried out. Optimum surfactant/co-surfactant mixture was then selected based on aqueous stability and phase behavior analysis. Alkali and NPs were finally screened by conducting static and dynamic adsorption tests in combination with screened polymer and surfactant.

### 2.1 Brines

The formation water (FW) provided directly from Field A was used to saturate the cores for the oil displacement tests. The density and pH of the formation water were 1.1 g/cc and 6.75, respectively. Caspian seawater (SW) with a salinity of around 14000 ppm was used to prepare chemical solutions for respective tests. Table 3 shows the composition of both brines. Brines were filtered to eliminate waste and solid particles before the injection to avoid clogging the pores and inaccurate results.

**Table 3:** Composition of Caspian seawater and formation water.

Ions	Concentration (ppm)	
	Seawater	Formation water
Na^+^	3513.1	18902.0
Ca^2+^	400.8	3307.0
Mg^2+^	790.4	972.8
Cl^−^	6026.6	36159.0
(SO_4_)^2-^	3138.0	75.8
HCO_3_^−^	256.2	390.4
K^+^	87.6	276.0
CO_3_^2-^	36.0	390.4
*Total*	*14248.7*	*60083*.*0*

### 2.2 Crude oil

Crude oil from Field A was used for the surfactant screening phase and oil displacement tests. The oil has a viscosity of 8 cp at the reservoir temperature of 63 °C. The oil contains considerable amounts of asphaltenes (13 wt%) and paraffin (20 wt%). Moreover, the oil density under reservoir conditions is 787 kg/m^3^. Crude oil was filtered to remove minerals and rock particles.

### 2.3 Rock samples

Sandstone core samples from the Field A reservoir section were used for chemical adsorption studies and oil displacement tests. Table 4 presents the physical properties of the cores used for oil displacement tests.

**Table 4:** Physical properties of the cores used for oil displacement tests.

Test	Sample ID	Length (cm)	Diameter (cm)	PV (cm^3^)	Porosity (%)	Permeability (mD)
Polymer Flooding (PF)	1	5.55	3.79	15.2	24.3	323.1
Nano-Polymer (NP) Flooding	2	5.57	3.78	15.6	25.0	422.2
Sequential Surfactant-Polymer (SP) Flooding	3	5.61	3.77	15.9	25.4	460.9
Combined Surfactant-Polymer (SP) Flooding	4	5.43	3.79	14.7	24.1	314.5
Alkali-Surfactant-Polymer (ASP) Flooding	5	5.67	3.78	15.5	24.4	355.7
Nano-Surfactant/Polymer (NSP) Flooding	6	5.65	3.79	16.2	25.4	241.3
Nano-Surfactant/Nano-Polymer (NS/NP) Flooding	7	5.31	3.78	15.0	25.2	294.5

The cores were manually saturated with formation water using a vacuum saturator to determine the samples' pore volumes (PV) using Equation (1).

(1) $$PV=\frac{W_{wet}-W_{dry}}{\rho_{FW}}$$

where *W*_*dry*_ is the dry weight of the core, *W*_*wet*_ is the wet weight of the core after saturation, and *ρ*_*FW*_ is the density of the formation water.

### 2.4 Chemicals

The chemicals assessed in this study for Field A EOR design included polymer, surfactant, alkali, and NPs. The chemicals screening stage examined several potential compounds utilizing various static methodologies and screening criteria through careful investigation and evaluation. A brief description of each chemical and its screening procedure is presented hereafter.

### 2.5 Polymer

The Field A operator supplied four hydrolyzed polyacrylamide (HPAM) based polymers. Samples were screened, and the optimum polymer was selected based on thermal stability, resistance to bacterial attack and oxygen, and static adsorption potential. The detailed procedure for the polymer selection stage can be found elsewhere [1]. The polymer solution was prepared in accordance with API 63 standard procedure. A 75% vortex was established in the makeup brine using a magnetic stirrer at a high speed of 600 rpm. Within 30 s, the dry polymer was continuously added to the vortex border. Immediately after that, the stirrer was set at a low speed (150 rpm) to prevent solid particles from coagulating and avoid degradation of the polymer solution. The solution was stirred gently for 2–3 h before letting it sit overnight. Parafilm was used to cover all the samples to minimize evaporation and potential errors in the results.

### 2.6 Surfactant

Several surfactants/co-surfactants supplied by the Field A operator were tested to screen the best surfactant based on aqueous stability, phase behavior, and IFT analysis. The surfactant selection has already been completed, and the results can be found in a recent study by our team [54]. To prepare surfactant solutions, the required amount of surfactant and/or co-surfactant was carefully weighed and slowly added to SW at a constant stirring speed of 200 rpm to prevent foaming in the solution. The solution was stirred for 1–2 h to ensure proper dissolution of the surfactant molecules in the brine. The solutions were sealed with parafilm and stored in a dry place to avoid contamination.

### 2.7 Alkali

To select the alkali for the ASP test, the alkalis sodium hydroxide (NaOH) and sodium carbonate (Na_2_CO_3_) were assessed. These particular alkalis were selected based on their proven ability to reduce adsorption in similar research [[55], [56], [57]]. The method for the alkali screening stage can be found in our previous study [54,58]. For coreflood tests, the alkaline solution was first prepared by carefully weighing the required amount of alkali and slowly adding it to the distilled water at a constant stirring speed of 600 rpm. The solution was stirred for 20–30 min. The prepared alkaline solution was added to the freshly prepared surfactant solution to achieve the target concentration.

### 2.8 Nanoparticles

As supported by prior studies, silica NPs were applied due to their demonstrated effectiveness in mitigating surfactant adsorption [59]. SkySpring Nanomaterials supplied 99.5% pure spherical porous silica NPs. These NPs had a density of 2.4 g per cubic centimeter and a size of 10–20 nm. To select the optimum concentration of NPs for coreflood tests, static and dynamic adsorption studies were performed with screened surfactant and polymer. The objective of static and dynamic adsorption tests is to identify the concentration of NPs that helps to lower the adsorption of chemicals onto rock surface. SiO_2_ NPs were tested at two concentrations of 0.05 and 0.1 wt% for surfactant and polymer adsorption control. The optimum concentration was used for respective nanofluid-polymer and nanofluid-surfactant oil displacement tests. To prepare the nanofluid solution, the NPs at optimum concentration were added to Caspian seawater and stirred using a magnetic stirrer for 1 h. The obtained solution was then subjected to ultrasonic homogenization for another hour. Ultrasonic homogenization is a process in which high frequency ultrasound waves are used to generate strong cavitation and acoustic cyclosis in the liquid. This powerful mechanical activity guarantees the reduction of particle size and boosts the overall stability and consistency of the nanofluid. Subsequently, nanoparticle solution was added to other chemical solutions such as surfactant and polymer solutions to conduct tests on hybrid EOR methods.

### 2.9 Oil displacement tests

**Fig. 2:**
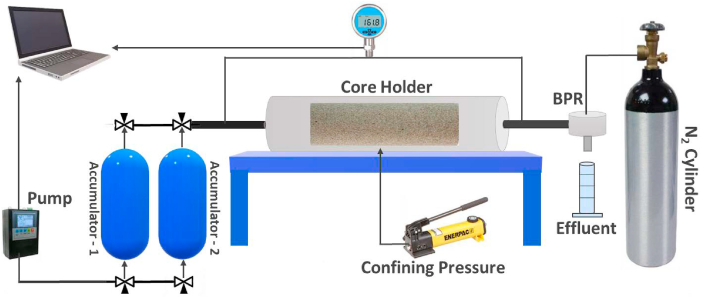
The schematic of coreflood system CFS-700 used for oil displacement tests.

Once the chemicals were selected based on systematic screening studies, oil displacement tests were conducted to evaluate the oil recovery potential of standalone and hybrid chemical flooding designs and select the best EOR scheme for Field A. CFS-700 core flooding system by Vinci Technologies, schematically shown in Fig. 2, was utilized to perform tests on core samples under reservoir conditions.

• The core sample was saturated with FW and then flooded with FW at different rates to determine the absolute permeability of the core. The CFS system was set under the reservoir conditions at a temperature of 63 °C and a confining pressure of 1500 psi. The back pressure was set at 300 psi throughout the test except during polymer injection. The back pressure during PF was set at 0 psi to avoid polymer degradation during outflow.
• Crude oil was then injected into the core at a flow rate of 0.5 cc/min and was continued until the effluent water cut was reduced to less than 0.1%. The injection rate was then raised to 1.0, 1.5, and 2.0 cc/min to overcome capillary end effects and reach initial water saturation (S_wi_) in the core. The conditions for switching the flow rate were to have an effluent water cut of less than 0.1% and a stabilized pressure drop across the core sample. The effluent water production data was utilized to calculate S_wi_ using Equation (2).

(2) $$S_{wi}=\frac{PV-V_{w}}{PV}\times 100$$

where PV is the pore volume of the core and *V*_*w*_ is the water volume collected in effluents.

• Next, the core was flooded with SW to recover oil by waterflooding. SW was injected at a rate of 0.5 cc/min until the oil cut in the effluent was less than 0.1%, and a stable pressure drop was maintained. The water injection rate was then increased to 1.0, 1.5, and 2.0 cc/min to minimize capillary end effects and to reach residual oil saturation after waterflooding (S_orw_). The volume of oil produced during waterflooding was used to calculate the recovery factor using Equation (3).

(3) $$Recovery\;factor=\frac{V_{oi}-V_{o}}{V_{oi}}*100$$

where $V_{oi}$ is the original oil in place (OOIP) and $V_{o}$ is the volume of oil produced by waterflooding.

• In the next step, the desired chemical solution at a particular concertation was prepared in SW and injected at 0.5 cc/min. The same criterion was observed to increase the flow rate to the next value. The volume of oil produced during this stage was used to calculate incremental recovery by chemical flooding.
• The values of the two-phase resistance factor (RF), residual resistance factor (RRF), and capillary number (N_c_) were calculated using Equations (4), (5), (6), respectively.

(4) $$Resistance\;Factor=\frac{{\Delta p}_{PF}}{{\Delta p}_{WF}}$$

(5) $$Residual\;Resistance\;Factor=\frac{{\Delta p}_{WF}}{{\Delta p}_{postflush}}$$

(6) $$N_{c}=\frac{K}{\sigma }{\left(\frac{\Delta p}{L}\right)}_{CF}$$

where *Δp*_*PF*_ is the pressure drop during polymer flooding, *Δp*_*WF*_ is the pressure drop during waterflooding, *Δp*_*postflush*_ is the pressure drop during SW postflush, *K* is absolute permeability, *(Δp/L)*_*CF*_ is the pressure gradient across the core during a specific chemical flooding stage and *σ* is the oil-brine IFT.

• Finally, a post-flush of SW was performed to calculate the residual resistance factor (RRF) using Equation (5) and to displace the un-adsorbed chemicals [60].

The same procedure was followed for all the oil displacement tests to identify the most suitable chemical flood design for Field A.

## 3 Results

### 3.1 Chemicals screening

**Fig. 3:**
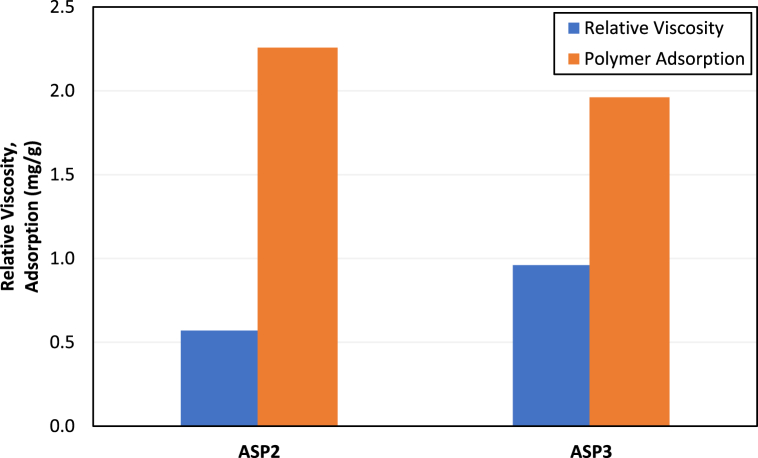
Comparison of relative polymer viscosity and polymer adsorption for ASP2 and ASP3.

The polymer was the first chemical constituent to be evaluated for the chemical flood design in this study. Out of four HPAM-based polymers, two polymers abbreviated as ASP2 and ASP3 retained the target viscosity of 5 cp at 2500 ppm concentration during long-term thermal stability analysis. These two polymers were then evaluated for their resistance to non-ionic material in SW, such as bacteria. Moreover, a static adsorption study was conducted to assess the adsorption tendency of the two polymers on the Field A rock surface [1]. Fig. 3 compares relative polymer viscosities after one-month aging at a reservoir temperature of 63 °C and static adsorption values for polymers APS2 and ASP3. The details of the polymer screening stage can be found elsewhere [1]. From the results presented in Fig. 3, polymer ASP3 at 2500 ppm concentration was selected as it showed around 14% lower adsorption than ASP2, and its resistance to bacterial activity was also 1.7 times higher than ASP2. A lower adsorption and higher resistance to bacterial activity ensures the target polymer concentration will be maintained during injection into the reservoir.

**Fig. 4:**
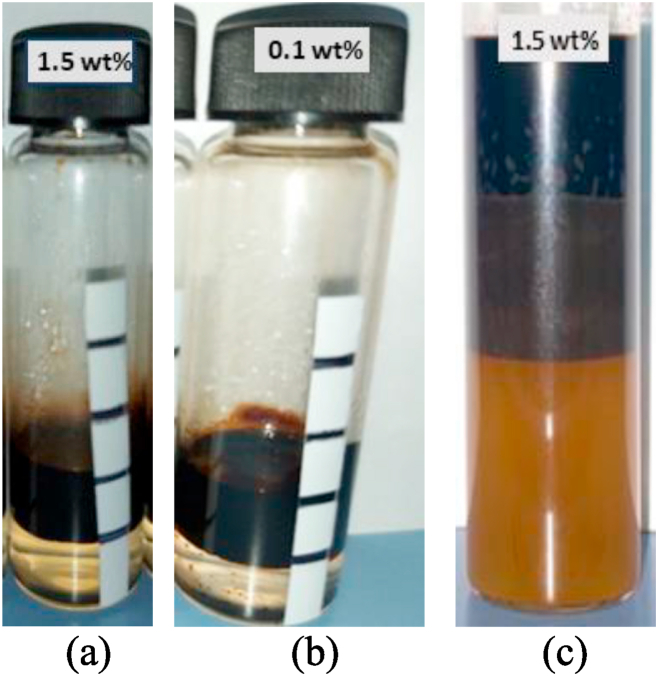
Phase behavior results for (a) 1S, (b) 2S, and (c) 2S/2A at surfactant/co-surfactant ratio of 30/70.

The surfactant screening stage for Field A consisted of assessing a total of 42 surfactant/co-surfactant mixtures for aqueous stability. The solutions that passed the aqueous stability test included surfactant 1S at concentrations above 0.1 wt%, surfactant Soloterra labeled as 2S at 0.1 wt% concentration, and surfactant/co-surfactant mixtures of surfactant Soloterra (2S) and co-surfactant Alfoterra (2A) at overall concentration in the range of 0.5–2.0 wt%. The stable surfactant solutions were then evaluated for Winsor Type III microemulsion generation by performing phase behavior with Field A crude oil. Fig. 4(a), b, and 4c show the phase behavior results for 1S, 2S, and 2S/2A samples, respectively. Winsor Type III microemulsions are important in EOR as they recover residual oil by achieving ultra-low IFT, augmenting oil displacement, and exhibiting compatibility with reservoir conditions. These characteristics make them a beneficial tool in exploiting the extraction of oil from problematic reservoirs.

It can be seen from Fig. 4 that only 2S/2A solution resulted in the generation of Winsor Type III microemulsion, which is favored for recovering residual oil. Different formulations of 2S/2A were then screened by IFT measurements to select the optimum surfactant formulation for further analysis. Table 5 presents IFT results for 1.5 wt% 2S/2A solution as a function of surfactant/co-surfactant ratio. The IFT analysis revealed that two orders of magnitude reduction in IFT were obtained by 1.5 wt% 2S/2A solution at a surfactant/co-surfactant ratio of 30/70. Consequently, the combination of Soloterra and Alfoterra at a mixing ratio of 30/70 and an overall concentration of 1.5 wt% was selected for the chemical flood design for Field A. The detailed procedure for surfactant screening has been reported elsewhere [54].

**Table 5:** IFT results for 1.5 wt% 2S/2A samples.

2S/2A Ratio	*IFT (mN/m)*
0	14.0
10/90	2.82
20/80	1.26
30/70	0.20
40/60	3.36
50/50	4.40

**Fig. 5:**
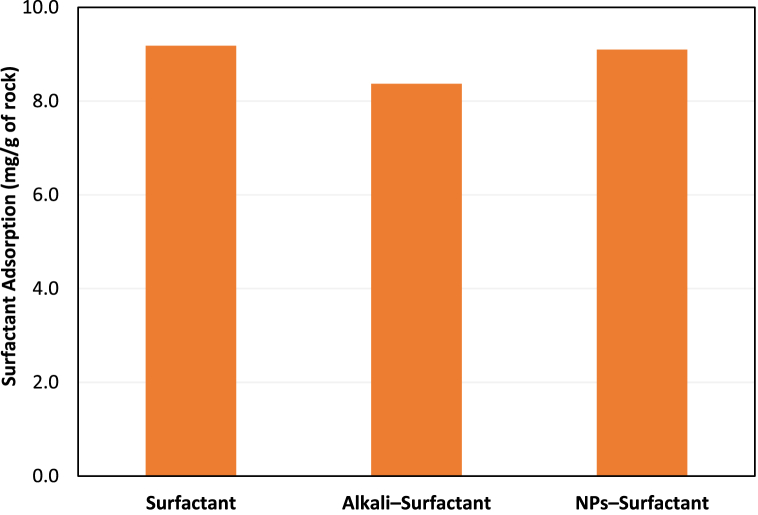
Surfactant dynamic adsorption in the presence of 0.02 wt% Na_2_CO_3_ and 0.05 wt% silica NPs.

The next step in the chemical screening phase was to select suitable alkali and NPs to lower surfactant adsorption. The details of this phase have been reported elsewhere [54]. The stable alkali and silica NPs solutions, which comprised 0.02 wt% NaOH, 0.02 wt% Na_2_CO_3_, and 0.05 and 0.1 wt% SiO_2_ NPs, were mixed with 2S/2A solution at optimum concentration and were observed for reduction in surfactant adsorption. Based on static adsorption results, alkali Na_2_CO_3_ exhibited a higher tendency to lower surfactant adsorption than NaOH. Likewise, silica NPs at a concentration of 0.05 wt% caused a more significant reduction in surfactant adsorption. The screened alkali and NPs were then employed during dynamic surfactant adsorption to assess their performance under realistic oilfield conditions. Fig. 5 shows the results of dynamic adsorption of surfactant in the presence of alkali and silica NPs. It is clear from Fig. 5 that alkali performed better than NPs in lowering surfactant adsorption under dynamic conditions. In principle, dynamic adsorption results provide a more inclusive understanding of how chemicals perform in the challenging and dynamic environments of reservoirs. This information is vital for developing efficient chemical solutions that address challenges and enhance oilfield operations. Based on combined static and dynamic adsorption analysis results, 0.02 wt% Na_2_CO_3_ was chosen as the optimum alkali for the ASP coreflood test, while 0.05 wt% silica NPs were selected for NSP coreflood.

**Fig. 6:**
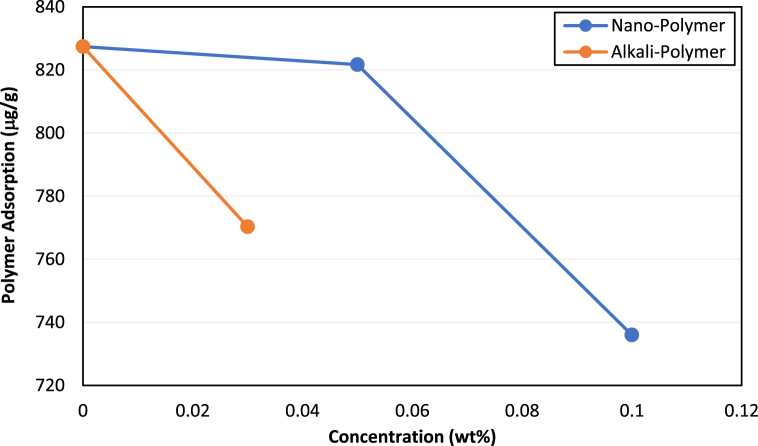
ASP3 polymer adsorption in the presence of alkali and silica NPs.

A similar adsorption study was then conducted to select the optimum alkali and NPs for controlling polymer adsorption. For this purpose, alkali NaOH at a stable concentration of 0.03 wt% and silica NPs at 0.05 and 0.1 wt% concentrations were analyzed for static adsorption control of ASP3 at optimum concentration. Fig. 6 shows the comparison of static adsorption of polymer for different conditions.

More details of the polymer adsorption control tests have already been presented in the study conducted by Maratbekkyzy et al. [58] NPs at a higher concentration caused more reduction in polymer adsorption, causing an 11% decrease in adsorption. NaOH also lowered polymer adsorption, resulting in a 7% decrease. The two best concentrations of alkali and NPs were then used to determine the dynamic adsorption of the polymer. Silica NPs at 0.1 wt% concentration exhibited a greater tendency to inhibit polymer adsorption under dynamic conditions than alkali. Almost 18% less polymer was adsorbed in the presence of NPs during the dynamic adsorption test. As a result, 0.1 wt% concentration of silica NPs was decided for the nano-polymer oil displacement tests, while alkali was discarded as an additive for polymer solutions. The reason for better performance of silica NPs in controlling polymer adsorption can be the sacrificial adsorption of NPs onto rock surface, thereby reducing the surface area available for polymer adsorption. Moreover, NPs improve polymer chains elongation and stability of polymer solutions, leading to reduction in polymer adsorption.

### 3.2 Oil displacement tests

After screening all the chemicals for Field A, several combinations of surfactant, polymer, alkali, and NPs were developed to test for enhanced oil recovery potential. The primary objective of these oil displacement tests is to evaluate the effectiveness of various chemical combinations in enhancing oil recovery from Field A. Table 6 presents the injection sequence and fluid compositions for the seven corefloods designed for Field A. The oil displacement tests aimed to quantify the synergy among the screened chemicals and select the optimum combination capable of lowering the residual oil saturation and yielding an efficient displacement front.

**Table 6:** Coreflood design and injection schemes for oil displacement tests under different scenarios.

Experiment ID/Name	Injection Sequence/Design	Remarks
Polymer Flooding (PF)	SW → Polymer → SW-Postflush	ASP3 Polymer selected from the screening study was injected at an optimum concentration of 2500 ppm.
Nano-Polymer (NP) Flooding	SW → Nano-Polymer → SW-Postflush	SiO_2_/ASP3 was injected at 0.05 wt % of NPs and 2500 ppm of polymer.
Sequential Surfactant-Polymer (SP) Flooding	SW → Surfactant → Polymer → SW-Postflush	1.5 wt% Soloterra and Alfoterra at 30/70 ratio as surfactant and 2500 ppm ASP3 as polymer were injected.
Combined Surfactant-Polymer (SP) Flooding	SW → Surfactant-Polymer → SW-Postflush	A combined solution of 1.5 wt% surfactant 2S/2A at a 30/70 ratio and 2500 ppm ASP3 polymer was injected.
Alkaline-Surfactant- Polymer (ASP) Flooding	SW → Alkali-Surfactant → Polymer → SW-Postflush	A combined solution of 1.5 wt% surfactant 2S/2A at 30/70 ratio and 0.02 wt% Na_2_CO_3_ alkali was injected after WF, followed by 2500 ppm ASP3 injection.
Nano-Surfactant/Polymer (NSP) Flooding	SW → Nano-Surfactant → Polymer→ SW-Postflush	A combined solution of 1.5 wt% surfactant 2S/2A at 30/70 ratio and 0.05 wt% silica NPs was injected after WF, followed by 2500 ppm ASP3 injection.
Nano-Surfactant/Nano-Polymer (NS/NP) Flooding	SW → Nano-Surfactant → Nano-Polymer → SW-post flush	A combined solution of 1.5 wt% surfactant 2S/2A at 30/70 ratio and 0.05 wt% silica NPs was injected after WF, followed by 2500 ppm ASP3 polymer and 0.1 wt% silica NPs injection.

These seven coreflood tests were designed with the aim to optimize polymer flooding performance by incorporating the synergistic mechanisms of combining polymer with surfactant, alkali, and nanoparticles. These synergistic effects include IFT reduction by surfactant, mobility control by polymer, reduced chemical adsorption by alkali and/or NPs, and enhanced polymer stability in presence of NPs [61,62]. Overall, these coreflood scenarios aim to assess the effectiveness of different chemical flooding strategies in enhancing oil recovery from Field A, considering factors such as interfacial tension reduction, mobility control, and alteration of rock wettability. The injection sequences and fluid compositions are designed to exploit synergies among the selected chemicals and optimize oil displacement efficiency.

### 3.3 Polymer flooding

**Fig. 7:**
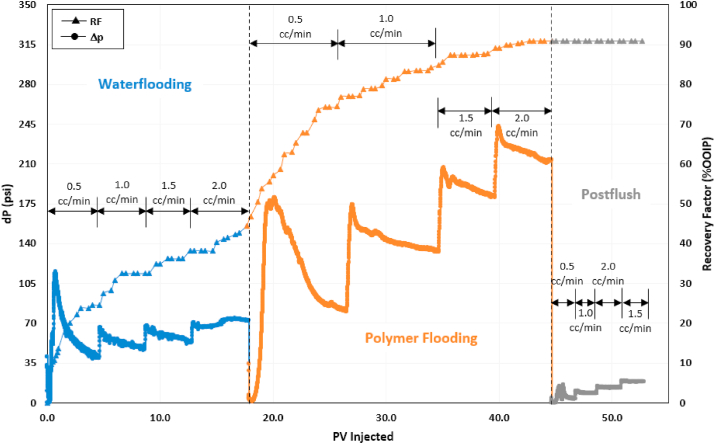
Recovery factor and pressure drop history of polymer flooding test.

The first oil displacement test in the series of corefloods was the injection of polymer ASP3. Fig. 7 shows the pressure drop and recovery factor data for this test. The experiment involved injecting SW to establish residual oil saturation after waterflooding (S_orw_). At the end of waterflooding, the recovery factor was around 43% of the original oil in place (OOIP). ASP3 polymer flooding was then initiated until no more oil was produced in the effluent. PF recovered 48% additional oil, increasing the total recovery factor to 91% OOIP. Finally, another post flush with seawater was performed to recover the un-adsorbed polymer and obtain pressure drop to calculate the residual resistance factor. The incremental recovery obtained during polymer flooding can be attributed to mobility control and effective oil bank displacement by ASP3 polymer.

#### 3.3.1 Nanofluid-polymer (NP) flooding

**Fig. 8:**
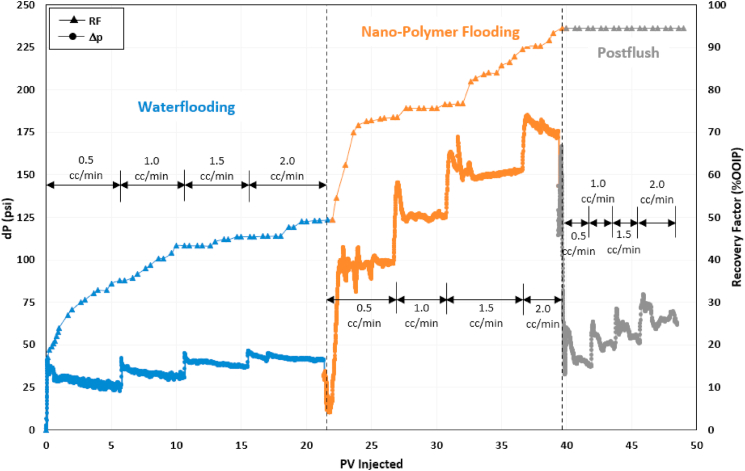
Recovery factor and pressure drop data of nanofluid-polymer (NP) flooding test.

The next oil displacement test was the injection of nanofluid-polymer. This test was similar to the polymer flooding test, except a mixture of 0.1 wt% silica NPs and ASP3 polymer was injected during the chemical flooding stage of this test. Fig. 8 presents the recovery factor and pressure drop data for the entire test. Waterflooding recovered 50% OOIP oil, followed by NP flooding, which resulted in 45% OOIP incremental oil recovery. Regarding remaining oil in the core (ROIC) after waterflooding, NP flooding produced 89% of ROIC, while PF recovered 85%. Thus, the incremental remaining oil recovery during NP flooding was 5% higher than that obtained during PF, and the pressure drop at each injection rate was relatively more stable.

The steady and significant pressure drop during nanofluid-polymer flooding is due to several factors. These include decreased polymer adsorption, enhanced polymer solution viscosity, and the synergistic actions of HPAM polymer and silica NPs, which may clog pores and divert flow. The pressure drop during the seawater postflush was 30–40% higher than during waterflooding. Several factors contribute to this increased postflush pressure drop. Firstly, it suggests that the polymer adsorption observed in this experiment is reversible, as the postflush water carries away un-adsorbed polymer. Secondly, there is the possibility that NPs and/or cross-linked polymer-NP structures may have obstructed certain pore throats, resulting in the heightened pressure drop during postflush.

### 3.4 Sequential and combined surfactant-polymer (SP) flooding

**Fig. 9:**
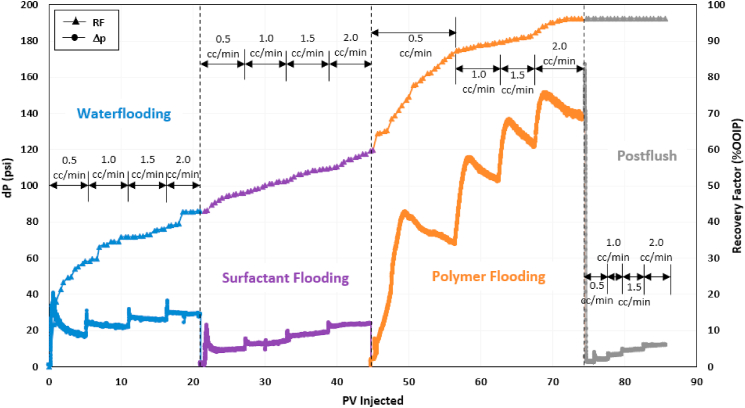
Recovery factor and pressure drop history of sequential surfactant-polymer (SP) flooding test.

**Fig. 10:**
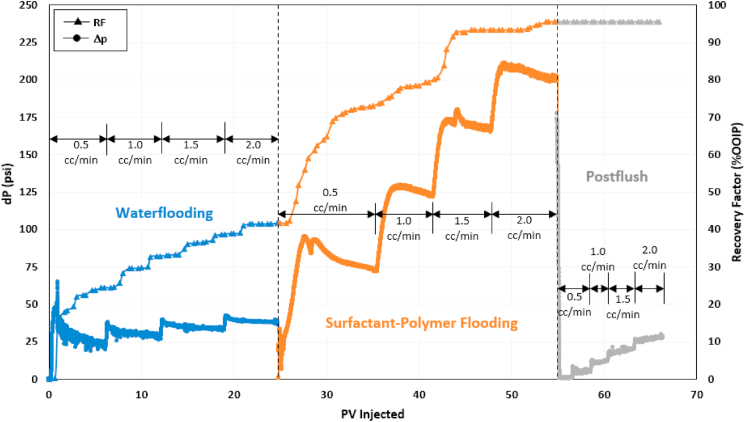
Recovery factor and pressure drop history of combined surfactant-polymer (SP) flooding test.

After evaluating polymer performance, the combination of surfactant and polymer was assessed for their synergy in recovering residual oil. The first surfactant-polymer test consisted of surfactant flooding in continuous mode, followed by polymer flooding. During the combined SP test, a combined solution of ASP3 polymer and 2S/2A surfactant at optimum concentration was injected. Fig. 9, Fig. 10 show the profiles of recovery factor and pressure drop for both tests. The recovery factor at the end of waterflooding during sequential SP flooding was around 43% OOIP, which later increased to 60% OOIP during surfactant flooding. Polymer flooding displaced an additional 36.4% OOIP, resulting in a total recovery factor of 96.2%. In combined SP flooding, 42% OOIP was produced during conventional waterflooding. The combined surfactant-polymer injection efficiently recovered the residual oil, producing 53% OOIP incremental oil.

**Fig. 11:**
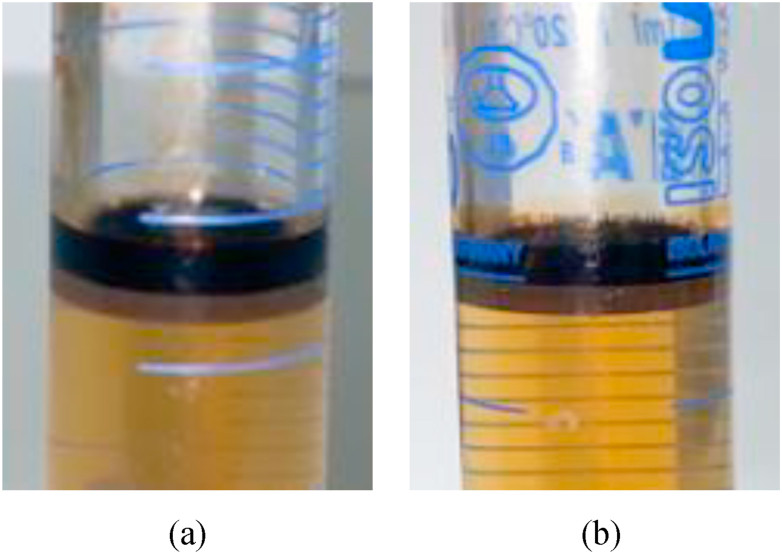
Winsor Type III microemulsion produced during (a) Sequential SP flooding and (b) Combined SP flooding.

Both sequential and combined SP flooding schemes generated Winsor Type III microemulsion in situ and successfully recovered more than 50% OOIP of oil left after waterflooding. Fig. 11(a) and b shows the microemulsion produced during the chemical flooding stage of both tests. These results indicate that both designs of SP flooding are equally efficient for microemulsion generation and IFT reduction.

Pressure drop fluctuations highlighted in Fig. 9, Fig. 10 can be attributed to unblocking of the pores by injected chemicals that were previously not contacted during waterflooding. It can be observed from sequential SP pressure performance that the surfactant solution efficiently reduced IFT as it came in contact with the trapped residual oil in blocked and unblocked pores. The microemulsion generated during surfactant injection was subsequently displaced by the viscous polymer.

In the case of combined SP flooding, it may take some time for the surfactant to reduce IFT and produce microemulsion, as evident by a delay in oil production during SP flooding in Fig. 10. However, the obvious benefit of combined SP design is that it requires almost 50% less chemical volume than sequential SP. These results show that combined SP flooding is preferable for the same concentrations of surfactant and polymer. Moreover, the pressure drop behavior demonstrates that combined SP performed better since it exhibited a higher pressure drop than that observed during the polymer flooding stage of the sequential SP flooding test. A high-pressure drop during combined SP flooding can be due to a high residual oil saturation experienced by the chemical solution. Due to this high-pressure drop, the injected SP solution unblocked a greater fraction of the pore space and contacted a higher saturation of residual oil. As a result, the incremental recovery by the combined method was slightly higher than the sequential design.

### 3.5 Alkaline-surfactant-polymer (ASP) flooding

**Fig. 12:**
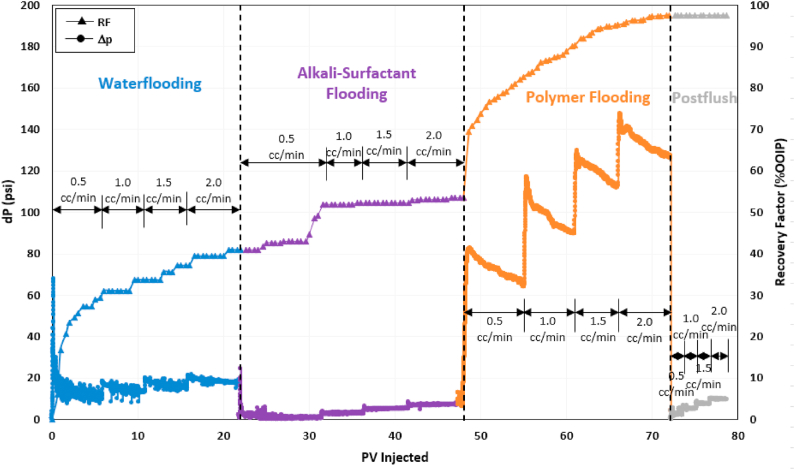
Recovery factor and pressure drop history of alkaline-surfactant-polymer (ASP) flooding test.

An alkaline-surfactant-polymer flooding test was performed to evaluate the synergy between selected alkali and surfactant. Fig. 12 presents the recovery factor and pressure drop trend for the entire test. After reaching S_orw_, the recovery factor by waterflooding was 41% OOIP. A mixture of 0.02 wt% Na_2_CO_3_ and 1.5 wt% 2S/2A at an optimum ratio of 30/70 was injected in continuous mode during the alkaline-surfactant (AS) flooding stage, increasing the recovery factor by an additional 13% OOIP. This incremental recovery can be recognized as the beneficial effect of alkali Na_2_CO_3_ in reducing surfactant adsorption, allowing more surfactant molecules to contact and emulsify the oil components. The initial increase in oil recovery within the first few pore volumes was relatively gradual, as it took time for the surfactant solution to interact with the oil and create microemulsions. Furthermore, at an injection rate of 0.5 cc/min, the pressure drop during AS flooding was considerably lower, which may not have been adequate to mobilize the oil and generate microemulsions as effectively as observed at higher flow rates. AS flooding had a pressure drop three to five times lower than seawater flooding, showing that alkali reduced surfactant adsorption and helped in maintaining high surfactant mobility.

Once there was no more oil production by AS flooding, the core was flooded with ASP3 polymer solution. PF exhibited a remarkable performance as the total recovery factor increased to 97.6%. Polymer flooding recovered an additional 44% of OOIP, and almost 95% of the remaining oil left after AS flooding. Such high incremental oil recovery by polymer is due to the combined effect of the ASP constituents. Alkali and surfactant helped to mobilize the trapped oil by lowering oil-water IFT and emulsifying the oil, whereas polymer provided the necessary viscous force to displace the mobilized oil. Thus, alkali-surfactant improved the microscopic sweep efficiency while polymer aided in increasing the macroscopic sweep efficiency. The pressure drop during water post-flush was almost 50% lower than the seawater flooding stage, indicating that almost all the residual oil had been recovered.

### 3.6 Nanofluid-surfactant/polymer (NSP) flooding

**Fig. 13:**
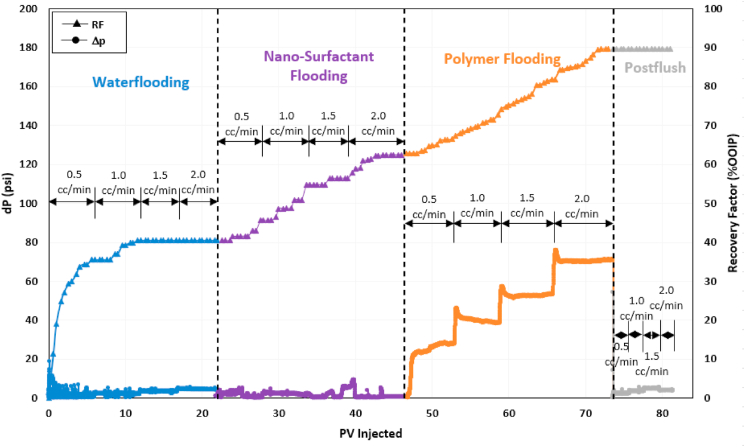
Recovery factor and pressure drop history of nano-surfactant/polymer (NSP) flooding test.

In addition to alkali, silica NPs also demonstrated encouraging results in lowering surfactant adsorption. To further evaluate the potential benefits of reduced chemical adsorption by NPs in terms of an increase in residual oil recovery, a nanofluid-surfactant/polymer flooding test was conducted. The pressure drop and recovery factor profiles for the entire test are depicted in Fig. 13. After reaching S_wi_, waterflooding was initiated, which recovered 40% of OOIP. The core was then flooded with a hybrid solution of 0.05 wt% silica NPs and 1.5 wt% 2S/2A at an optimum ratio of 30/70 in continuous mode. This injection stage resulted in an additional 22% recovery of oil.

The incremental oil recovery is linked to the surfactant's ability to reduce interfacial tension and the NPs' role in controlling adsorption. Additionally, the NPs might have contributed by obstructing select pores and redirecting the surfactant toward previously unexposed regions, as evidenced by the recurring variations in pressure drop during nanofluid-surfactant flooding. After the oil production by NPs-surfactant flooding decreased to almost zero, the core was continuously flooded with ASP3 polymer solution. PF recovered 27% OOIP additional oil, raising the total recovery factor to 90% OOIP. This incremental oil recovery can be attributed to the mobility control action of the polymer, the stable displacement front, and the production of the residual oil previously mobilized by the NPs-surfactant solution. Lastly, seawater post-flush was conducted to recover the un-adsorbed chemicals. The pressure drop during post-flush was almost similar to the pressure drop during seawater flooding, indicating a successful oil recovery performance of NSP flooding.

The similar recovery factors by polymer flooding and nano-surfactant/polymer flooding can be explained by the insignificant role of NPs in lowering surfactant adsorption. Since NPs were not as effective in minimizing surfactant adsorption, a lesser volume of microemulsion was generated during the NPs-surfactant flooding stage. Moreover, re-trapping of some oil droplets could also lead to a lower incremental oil recovery by subsequent polymer flood. Thus, there was no additional improvement in remaining oil recovery by nano-surfactant/polymer flooding if compared to polymer flooding.

Comparing the performance of the NSP test with that of ASP flooding, it can be seen that ASP design yielded better results and recovered more oil. Another observation from the pressure drop profiles of the two tests is that the alkali in the ASP flooding also lowered polymer adsorption as it made the rock surface more negatively charged. As a result, the injected polymer solution retained its viscosity better, which is evident by the polymer's almost two times higher pressure drop in ASP compared to NSP.

### 3.7 Nanofluid-surfactant/nanofluid-polymer (NSNP) flooding

**Fig. 14:**
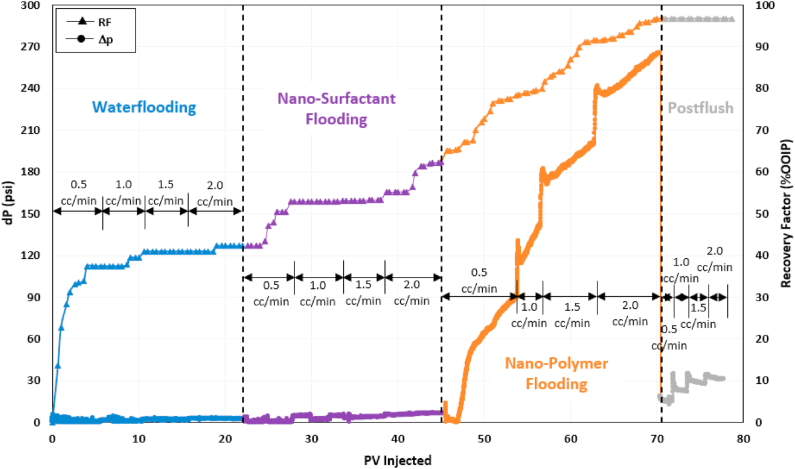
Recovery factor and pressure drop history of nanofluid-surfactant/nanofluid-polymer (NSNP) flooding test.

The objective of this test was to compare the performance of the NSNP test with the NSP flooding test. The pressure drop and recovery factor trend for the NSNP test is shown in Fig. 14. SW flooding recovered 42% OOIP while the nanofluid-surfactant (NS) stage produced 20% OOIP additional oil, similar to the nanofluid-surfactant recovery of the NSP test. The main difference between the NSP and NSNP tests is observed during the polymer injection stage. Due to the addition of NPs, the polymer flooding stage of the NSNP test recovered 34% OOIP oil, almost 7% more compared to the polymer flooding recovery of the NSP test. This increase in oil recovery by nanofluid-polymer is due to the lower polymer adsorption and two times increase in polymer in-situ viscosity. Part of this incremental recovery can also be due to pore blockage by NPs, as evidenced by a steady increase in pressure drop over time during nanofluid-polymer flooding. Due to high viscous forces and elevated pressure drop, the injected polymer solution is diverted to inaccessible pore volume, thereby contacting a higher percentage of remaining oil and efficiently recovering the residual oil bank created by the previously injected nanofluid-surfactant solution.

### 3.8 Comparison of oil displacement efficiency of various EOR designs

Having performed the coreflood tests with different chemical design scenarios, the oil recovery efficiencies of all the flooding tests were compared to narrow down to the best/practical chemical flooding recipe for the field. Table 7 summarizes the recovery factors obtained during different injection stages of each coreflood test. All seven enhanced oil recovery tests demonstrate that secondary recovery was nearly identical for all cores. This outcome was expected since the cores were chosen from the same formation and possessed similar reservoir rock characteristics.

**Table 7:** Summary of oil recovery factors for different chemical flooding designs.

Test Type	Flooding Stage	Recovery Factor	Incremental RF	
		(%OOIP)	(%OOIP)	(%ROIC)
Polymer Flooding	SW	42.8	–	–
	Polymer	90.9	48.2	84.1
Nano-Polymer Flooding	SW	49.5	–	
	Nano-Polymer	94.5	45.0	89.2
Sequential Surfactant-Polymer Flooding	SW	42.9	–	
	Surfactant	59.8	16.9	29.6
	Polymer	96.2	36.4	90.6
Combined Surfactant-Polymer Flooding	SW	41.8	–	
	Surfactant-Polymer	95.5	53.8	92.3
Alkali-Surfactant-Polymer Flooding	SW	41.0	–	
	Alkali-Surfactant	53.5	12.6	21.3
	Polymer	97.6	44.0	94.7
Nano-Surfactant/Polymer Flooding	SW	40.5	–	–
	Nano-Surfactant	62.5	22.0	37.0
	Polymer	89.7	27.1	72.4
Nano-Surfactant/Nano-Polymer Flooding	SW	42.3	–	
	Nano-Surfactant	62.4	20.1	34.8
	Nano-Polymer	96.8	34.4	91.4

Similar recovery factors in polymer flooding and nano-surfactant/polymer flooding can be attributed to NPs playing an insignificant role in reducing surfactant adsorption. The limited effectiveness of NPs in minimizing surfactant adsorption resulted in a smaller volume of microemulsion during the NPs-surfactant flooding stage. Additionally, the re-trapping of some oil droplets contributes to a lower incremental oil recovery in subsequent polymer flooding stage. Consequently, nano-surfactant/polymer flooding did not yield additional improvement in remaining oil recovery compared to polymer flooding.

The main recovery mechanism is attributed to the combined use of surfactant and polymer. These results highlight the efficacy of several enhanced oil recovery approaches analyzed in this study, with incremental recovery factors for various flooding schemes ranging from 36% to 54%. Among all coreflood tests, ASP flooding proved to be the best EOR design to improve remaining oil recovery, as it successfully produced 96% ROIC after waterflooding.

**Fig. 15:**
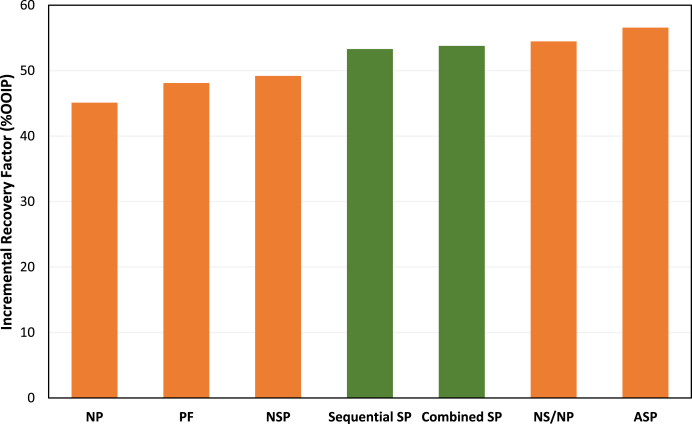
Comparison of incremental oil recovery from Field A by various EOR designs.

Fig. 15 visually compares incremental oil recovery factors for different cases. The application of NP and alkali can affect the recovery due to the adsorption control, but the difference with the conventional SP flooding is about 2%, which is not noticeable. Due to the difficulties of handling additional chemicals at the field scale, the application of SP flooding is recommended for this case.

**Fig. 16:**
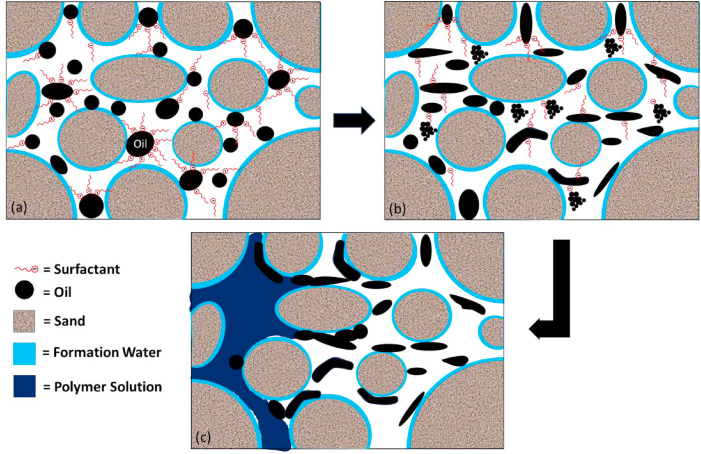
Schematic illustration of mechanisms responsible for EOR by SP flooding; (a) Action of surfactant molecules on oil-water interface (b) Oil-water IFT reduction by surfactant and generation of microemulsion, and (c) Accumulation and displacement of released oil droplets by polymer solution.

The mechanisms responsible for the recovery of residual oil by SP flooding are schematically shown in Fig. 16(a–c). Surfactants play a role in reducing interfacial tension and facilitating the separation of oil from rock. Concurrently, polymers elevate the viscosity of the solution, enhancing both the sweep efficiency and conformity of the injected fluid. The collaborative impact of these mechanisms fosters capillary desaturation, facilitating a more effective displacement of oil within the reservoir. Furthermore, SP flooding is acknowledged for alleviating undesirable effects, such as gravity segregation and viscous fingering.

Our study showed that the effect of polymer is dominant in the performance of the chemical EOR techniques in our case. To evaluate the performance of ASP3, RF values are compared, as shown in Table 8. RF is calculated by comparing the stabilized pressure drop during chemical flooding to that during water flooding (pre-flush).

**Table 8:** Resistance factors for the oil displacement tests.

Test Type	Resistance Factor
Polymer Flooding	2.77
Nanofluid-Polymer Flooding	4.07
Sequential SP Flooding	4.57
Combined SP Flooding	4.45
Alkali-Surfactant-Polymer Flooding	6.83
Nanofluid-Surfactant/Polymer Flooding	17.38
Nanofluid-Surfactant/Nanofluid-Polymer Flooding	134.22

It can be seen from Table 8 that polymer flooding reduced the mobility of all cases. The change is more by applying nanofluid and alkali, which is due to the presence of more polymer in the solution. The mobility ratio reduction during PF and SP seems enough to achieve a high recovery factor. Adding chemicals such as NPs and alkali increases RF but is practically unnecessary.

**Fig. 17:**
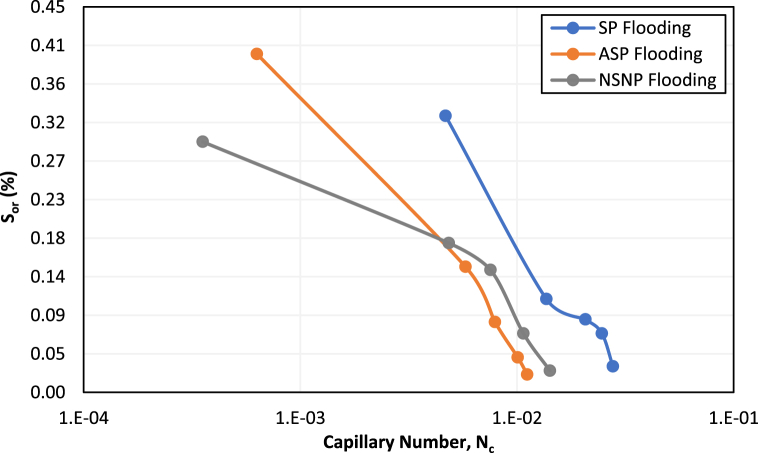
Capillary desaturation curves for SP, ASP, and NSNP coreflood tests.

Fig. 17 shows the capillary desaturation curves for surfactant-polymer, alkali-surfactant-polymer, and nanofluid-surfactant/nanofluid-polymer tests. It can be observed from Fig. 17 that for a similar range of capillary numbers, ASP flooding yielded a lower residual oil saturation compared to the other two methods at the lab scale. The crossover between NSNP and ASP capillary desaturation curves at a capillary number of 5x10^−3^ can be explained by an increase in viscous forces in the NSNP flooding test. Since NPs cause an increase in polymer viscosity, during the nano-polymer flooding stage of the NSNP test, the magnitude of viscous forces was higher owing to a higher pressure drop. This resulted in higher capillary numbers by NSNP flooding after the crossover point while the corresponding reduction in S_or_ was almost the same for both designs. Therefore, the reversal in residual oil saturation yielded by NSNP and ASP can be explained by increased viscous forces in the nano-polymer flooding stage of NSNP and enhanced emulsifying ability with ultra-low interfacial tension during the alkali-surfactant flooding stage of ASP.

Consequently, during nano-polymer flooding of the NSNP test, although the capillary number became higher due to increased viscous forces, the reduction in residual oil saturation was less due to lower volume of microemulsion generated during nano-surfactant injection stage and re-trapping of some released oil drops. On the contrary, during the polymer flooding stage of the ASP test, the capillary number was slightly lower than the NSNP test but the reduction in S_or_ was higher due to efficient IFT reduction and microemulsion generation by alkali-surfactant which was then displaced by polymer front. Overall, the reduction in S_or_ by ASP flooding was 5–6% more than the SP and NSNP schemes. As mentioned before, this difference is not encouraging for the field application.

## 4 Conclusion

This study evaluated the performance of carefully selected chemicals, including polymer, surfactant, alkali, and NPs, on improving sweep efficiency and recovering residual oil from a waterflooded field in Kazakhstan. To quantify the gain in oil production realized as a function of different combinations of screened chemicals, seven coreflood tests were conducted using target field reservoir cores.

Among all the EOR designs evaluated, the incremental recovery factor by ASP flooding, at the lab scale, was the maximum, around 57% OOIP, about 3% higher than SP flooding. The main mechanism behind this improved recovery can be attributed to the generation of emulsions and the facilitation of easier oil production by improved mobility and sweep efficiency of the injected fluid by the polymer. The application of nanofluid to control the adsorption of polymer/surfactant was also successful at the lab scale, which was observed by the alteration in mobility. The recovery factor by this hybrid method was close to SP flooding.

The introduction of alkali and nanofluid into the chemical flooding process appears to have contributed to the formation of stable emulsions and reduction in chemical adsorption. The increase in recovery compared to the SP flooding was not high enough to convince the operators to use more complicated ASP flooding at the field scale, and surfactant-polymer flooding was selected for further pilot tests.

## Data availability statement

Data will be made available on request.

## CRediT authorship contribution statement

**Mariam Shakeel:** Writing – review & editing, Methodology, Formal analysis. **Dilyara Sagandykova:** Writing – original draft, Methodology, Formal analysis, Data curation. **Aibek Mukhtarov:** Formal analysis, Data curation. **Amina Dauyltayeva:** Formal analysis, Data curation. **Laila Maratbekkyzy:** Formal analysis, Data curation. **Peyman Pourafshary:** Writing – review & editing, Supervision, Methodology. **Darya Musharova:** Validation, Supervision.

## Declaration of competing interest

The authors declare that they have no known competing financial interests or personal relationships that could have appeared to influence the work reported in this paper.
